# The influence of murine cytomegalovirus infection on susceptibility to mycobacterial infection

**DOI:** 10.1016/j.isci.2026.115518

**Published:** 2026-03-27

**Authors:** Shuailin Li, Claire Hutchings, Marcellus Korompis, Christopher De Voss, Alberta Ateere, Iman Satti, Paul Klenerman, Helen McShane, Elena Stylianou

**Affiliations:** 1 The Jenner Institute, University of Oxford, Oxford OX3 7DQ, UK; 2 Peter Medawar Building for Pathogen Research and Translational Gastroenterology Unit, Nuffield Department of Clinical Medicine, University of Oxford, Oxford OX1 3SY, UK

**Keywords:** disease, immunology, bacteriology, viral microbiology

## Abstract

Cytomegalovirus (CMV) infection has been associated with an increased risk of tuberculosis, but the underlying mechanisms remain unclear. Here, we used the murine CMV (MCMV), a virus genetically and biologically related to human CMV, to interrogate potential mechanisms. MCMV-infected macrophages showed reduced expression of CD80, CD86, and MHC class II, decreased phagocytosis of mycobacteria, and enhanced mycobacterial killing compared with MCMV-uninfected macrophages. Splenocytes from MCMV-infected mice better controlled mycobacterial growth *ex vivo* than those from MCMV-uninfected mice. However*,* mice infected with MCMV and subsequently challenged intranasally with mycobacteria were not more susceptible to mycobacterial infection *in vivo*, irrespective of the tested MCMV infection route, the interval between infections, or Bacillus Calmette-Guérin (BCG) vaccination status. These findings offer insights into how prior CMV infection may modulate host responses to mycobacteria. Further studies using more virulent mycobacterial strains, more susceptible mouse models, and varied infection timings are needed to extend these results.

## Highlights

• MCMV downregulates the expression of CD80, CD86, and MHC class II on macrophages
• MCMV reduces mycobacterial phagocytosis but improves intracellular killing
• Splenocytes from MCMV-infected mice show better mycobacteria control *ex vivo*
• Prior MCMV infection does not increase susceptibility to mycobacterial infection *in vivo*

## Graphical abstract

**Figure:**
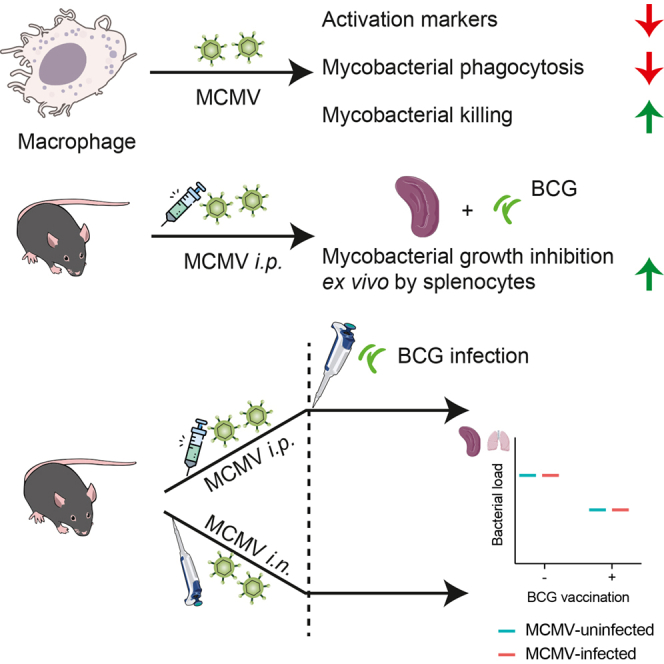

## Introduction

Tuberculosis (TB), caused by the bacterium *Mycobacterium tuberculosis* (Mtb), is the world’s leading cause of death from a single infectious agent.^1^ Cytomegalovirus (CMV) is a member of the β-herpesvirus family with double-stranded DNA, and it infects around 80%–90% of the world’s population.^2^ CMV infection is usually asymptomatic in immunocompetent individuals but can cause severe disease in immunocompromised individuals, such as those with HIV/AIDS and organ transplant recipients.^3^^,^^4^ In immunocompetent individuals, CMV can establish lifelong latency after primary infection, during which it may replicate chronically or reactivate sporadically.^5^ This sporadic viral activity appears to account for significant changes in the T cell phenotype of the infected individuals.^6^ This is supported by findings from a study on monozygotic twins where discordance in CMV infection affected more than half of the measured immune parameters, including those related to T cell phenotypes.^7^

CMV seropositivity rate is highly variable across different countries, with around 50% in UK adults but nearly 100% in those living in low- and middle-income countries, which are also TB-endemic areas, such as sub-Saharan Africa, India, and China.^1^^,^^8^ More than 60% of infants are infected with CMV in these areas by the age of 6 months.^3^^,^^8^^,^^9^^,^^10^ Several cohort and case-control studies have suggested that CMV infection during the first few months of life was associated with an increased risk of TB disease during follow-up and showed a trend toward a higher prevalence of Mtb infection following CMV infection.^11^^,^^12^^,^^13^ CMV-specific IgG levels in the blood have also been reported to be correlated with an increased risk of TB disease in adults.^14^^,^^15^ A comprehensive review has highlighted the complexity of the epidemiological and immunological relationship between CMV and TB; however, the mechanisms that account for the increased susceptibility to TB in CMV-infected individuals are less well understood.^16^

CMV has strict species specificity. Human cytomegalovirus (HCMV) cannot infect mice. However, murine cytomegalovirus (MCMV) infection in mice recapitulates features of HCMV infection in humans, such as memory inflation of CMV-specific T cells, pathogenesis, latency establishment, and broad tissue tropism.^16^^,^^17^^,^^18^ Thus, MCMV is widely used to study CMV biology. Breastfeeding in the early stages of life was found to be strongly correlated with CMV positivity in the nasopharyngeal swab of infants by 12 weeks, six months, and one year.^12^ We hypothesized that CMV may interact with Mtb at the mucosal surface in infants, particularly within macrophages, which can be infected by both CMV^19^ and Mtb.^20^ Such interactions at the mucosal site may further influence the susceptibility to mycobacterial infection.

In this study, we investigated the interaction between MCMV and mycobacteria *in vitro* and *in vivo* in mice. We found that prior MCMV infection reduced the expression of macrophage activation markers, decreased mycobacterial phagocytosis, and improved mycobacterial killing in distinct macrophage populations. However, *in vivo* MCMV infection did not increase susceptibility to mycobacterial infection, regardless of the tested routes of MCMV infection, the tested intervals between MCMV and mycobacterial infections, or the Bacillus Calmette-Guérin (BCG) vaccination status of mice.

## Results

### MCMV infection leads to reduced expression of activation markers in macrophages

Previous studies have shown that MCMV infection results in changes in cell surface protein expression in macrophages both *in vitro* and *in vivo.*^19^^,^^21^^,^^22^^,^^23^ These changes can, therefore, be used to distinguish MCMV-infected macrophages from bystander uninfected macrophages (exposed to MCMV but remaining uninfected). To investigate the impact of MCMV infection on macrophage activation marker expression, we used three macrophage types: bone marrow-derived macrophages (BMDMs), alveolar macrophages (AMs), and RAW 264.7, a macrophage cell line derived from a tumor in a male mouse induced by the Abelson murine leukemia virus.^24^

**Figure 1:**
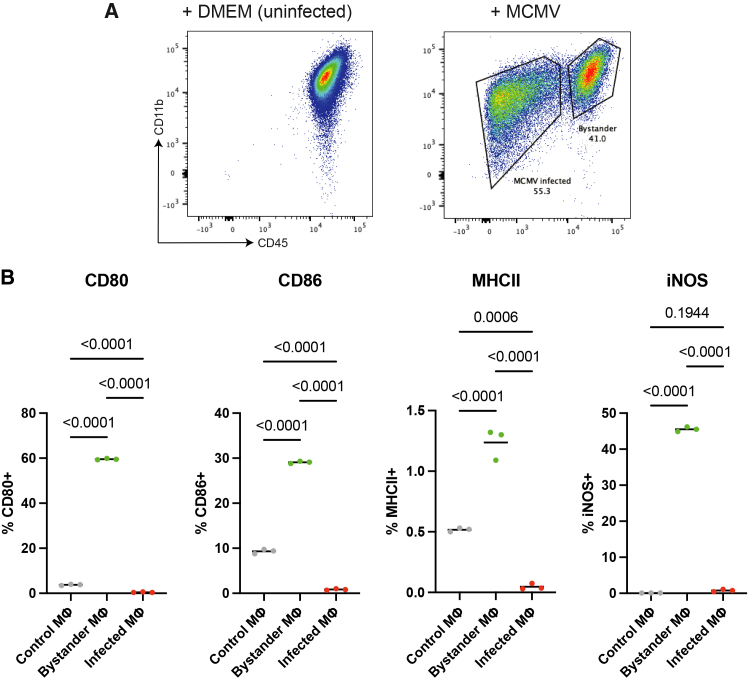
MCMV infection reduces the expression of cell surface and activation markers in macrophages (A) Representative flow cytometry plots showing the definition of uninfected (control), bystander, and MCMV-infected RAW 264.7 cells. RAW 264.7 cells were either left untreated (+DMEM) or treated with MCMV (+MCMV). (B) The percentage expression of CD80, CD86, MHC class II, and iNOS in uninfected or MCMV-infected and bystander RAW 264.7 cells. The line represents the mean value. One-way ANOVA with Tukey’s multiple comparison test was used to compare the expression of activation markers between different groups. Gray dots represent macrophages left untreated with MCMV, green dots represent bystander macrophages, and red dots represent MCMV-infected macrophages. *n* = 3 biological replicates per group. The multiplicity of infection (MOI) of MCMV used was 10. Flow cytometry was performed two days post-MCMV infection. See also Figures S1 and S2.

Previous work with MCMV-GFP demonstrated that infected and bystander RAW 264.7 cells, BMDMs, and AMs constitute two phenotypically distinct populations, identifiable by specific cell surface markers: CD45 for RAW 264.7 cells and BMDMs, and Siglec-F and CD11c for AMs.^19^^,^^21^ Using the same principles, we classified RAW 264.7 cells after MCMV exposure as MCMV-infected (CD45^−^) or bystander (CD45^+^) (Figures 1A and S1A). Similarly, BMDMs were classified as CD45^−^ (infected) or CD45^+^ (bystander), and AMs as SiglecF^−^CD11c^−^ (infected) or SiglecF^+^CD11c^+^ (bystander) (Figures S1B–S1F).

To investigate how MCMV infection affects macrophage activation, we compared the expression of activation markers in three different macrophage populations: (1) uninfected (not exposed to MCMV), (2) bystander (exposed to MCMV but not infected), and (3) infected (exposed to and infected with MCMV).

We measured the expression of CD86, CD80, MHC class II, and inducible nitric oxide synthase (iNOS) because of their roles in T cell activation and Mtb growth control.^25^^,^^26^ In RAW 264.7 cells, bystander macrophages significantly upregulated CD80, CD86, MHC class II, and iNOS compared with control and MCMV-infected macrophages (Figure 1B, *p* < 0.0001 for all comparisons). In contrast, MCMV-infected macrophages significantly downregulated CD80 (*p* < 0.0001), CD86 (*p* < 0.0001), and MHC class II (*p* = 0.0006) compared with control macrophages (Figure 1B). A similar pattern was observed in BMDMs, where bystander macrophages significantly upregulated CD80 (*p* < 0.0001 for both control and MCMV-infected macrophages), CD86 (*p* < 0.0001 for both control and MCMV-infected macrophages), and iNOS (*p* = 0.0008 and 0.0005 for control and MCMV-infected macrophages, respectively) compared with control and MCMV-infected macrophages, while MCMV-infected macrophages significantly downregulated CD86 (*p* < 0.0001) and MHC class II (*p* < 0.0001) compared with control macrophages (Figure S2A). In AMs, there was no expression of iNOS. However, CD80 (*p* = 0.001 and <0.0001 for control and MCMV-infected macrophages, respectively), CD86 (*p* < 0.0001 for both control and MCMV-infected macrophages), and MHC class II (*p* < 0.0001 for both control and MCMV-infected macrophages) levels were significantly higher in bystander than in control and MCMV-infected AMs (Figure S2B). The expression of MHC class II was downregulated in MCMV-infected AMs compared with that in control AMs (Figure S2B).

### MCMV infection reduces mycobacterial phagocytosis by macrophages

**Figure 2:**
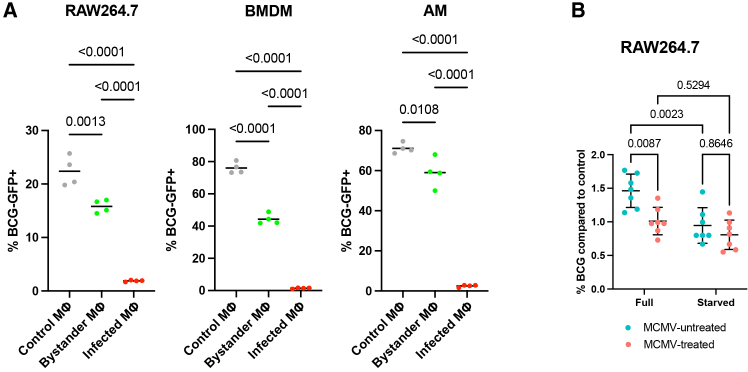
MCMV infection changes phagocytosis and killing of mycobacteria by macrophages (A) Phagocytosis rate of BCG-GFP in RAW 264.7 cells, BMDMs, and AMs after MCMV infection. The line represents the mean value. One-way ANOVA with Tukey’s multiple comparison test was used to compare phagocytosis of BCG-GFP between different populations. Gray dots represent macrophages left untreated with MCMV, green dots represent bystander macrophages, and red dots represent MCMV-infected macrophages. *n* = 4 biological replicates per group. (B) Survival of BCG in RAW 264.7 cells after MCMV infection. “Full” represents the survival of BCG in macrophages under full nutrition, and “starved” represents the survival of BCG in macrophages when autophagy is induced. The BCG survival was normalized to a control group of macrophages (MCMV-treated and MCMV-untreated) infected with BCG, which were harvested immediately after 1-h incubation to account for the difference in the phagocytosis rate. Two-way ANOVA with Tukey’s multiple comparison test was used to compare the survival of BCG between different conditions. The error bar indicates the mean value with standard deviation (SD). *n* = 7 biological replicates per group. The MOIs of MCMV were 10 for RAW 264.7 cells, 2 for BMDMs, and 1 for AMs. The phagocytosis and mycobacterial killing assays were performed two days post-MCMV infection. See also Figure S2.

MCMV infection of macrophages has been shown to inhibit phagocytosis of a variety of particles, including radiolabeled *Staphylococcus aureus*^27^^,^^28^ and *Legionella pneumophila (*Lp*)*.^19^ To investigate if MCMV infection influences the phagocytosis of mycobacteria, we infected macrophages with MCMV and measured the uptake of BCG-GFP following 4-h incubation. Compared with control macrophages, MCMV-infected and bystander macrophages had a significantly lower phagocytosis rate (Figure 2A, *p* < 0.0001 for MCMV-infected macrophages vs. control or bystander macrophages; *p* = 0.0013, < 0.0001, and 0.0108 for control vs. bystander macrophages in RAW 264.7 cells, BMDMs, and AMs, respectively). The reduction of the phagocytosis rate in bystander macrophages was moderate, whereas the phagocytosis rate in MCMV-infected macrophages was close to zero. These results indicated that MCMV infection could inhibit the phagocytosis of mycobacteria by macrophages, even in bystander cells.

### MCMV infection can enhance killing of mycobacteria by macrophages

To evaluate if MCMV infection influences mycobacterial killing by macrophages, RAW 264.7 cells, BMDMs, and AMs were infected with MCMV or left untreated, followed by infection with BCG at a multiplicity of infection (MOI) of 1. Given the role of autophagy in mycobacterial killing in macrophages,^29^^,^^30^ we also measured BCG killing under both baseline conditions and in response to autophagy induction to determine whether MCMV infection influences this process (see STAR Methods). Intracellular BCG survival was normalized to the phagocytosis rate. In MCMV-treated RAW 264.7 cells, BCG survival was significantly inhibited compared with that in untreated cells (Figure 2B, *p* = 0.0087). However, when autophagy was induced (starved), BCG survival was similar between the MCMV-treated and -untreated cells (Figure 2B). This suggests that MCMV infection impairs autophagy-mediated killing of BCG. No significant difference in the survival of BCG was observed in MCMV-treated and -untreated BMDMs or AMs (Figure S2C).

### MCMV infection improves mycobacterial control by splenocytes *in vitro*

**Figure 3:**
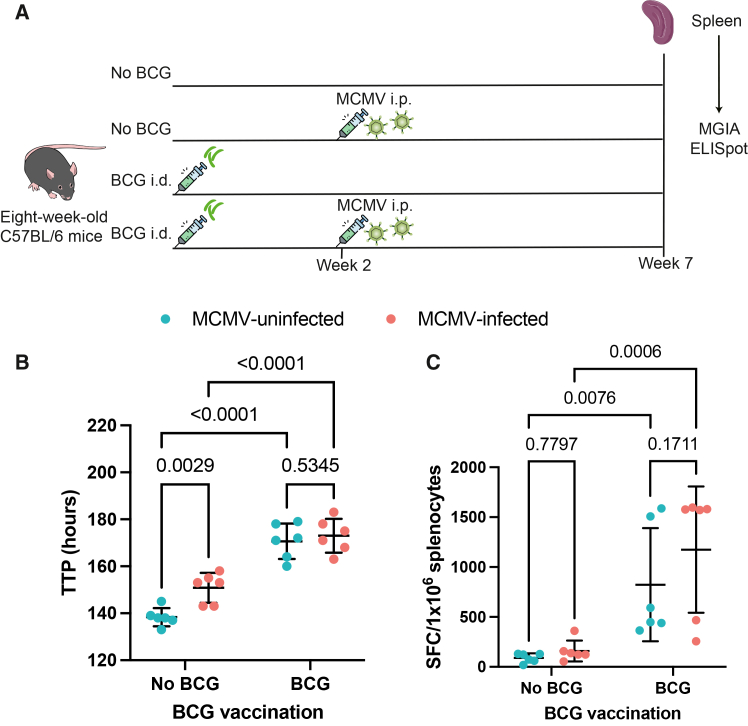
The influence of MCMV infection on the control of BCG growth by splenocytes *ex vivo* and on PPD-specific T cell response of splenocytes (A) Schematic of the experiment. (B and C) The TTP of BCG growth in the presence of splenocytes (B) and the PPD-specific T cell response of splenocytes (C) from BCG-unvaccinated MCMV-uninfected, BCG-unvaccinated MCMV-infected, BCG-vaccinated MCMV-uninfected, and BCG-vaccinated MCMV-infected mice. The PPD-specific T cell response is presented as the number of IFN-γ spot-forming cells (SFCs) per 10^6^ splenocytes. “No BCG” means no prior BCG vaccination of the mice. Red dots represent MCMV-infected mice. Blue dots represent MCMV-uninfected mice. Uncorrected two-way ANOVA was used to compare the TTP and PPD-specific T cell response between different groups under each condition. The error bar indicates the mean value with SD. *n* = 6 mice per group. TTP, time to positivity; PPD, purified protein derivative. See also Figure S3.

The mycobacterial growth inhibition assay (MGIA) is an *ex vivo* tool used to assess the protective efficacy of TB vaccines by testing the ability of cells from tissues, such as splenocytes, to control mycobacterial growth.^31^ To determine whether MCMV infection affects this response, mice were either vaccinated with BCG or left unvaccinated, with some groups subsequently infected with MCMV intraperitoneally (i.p.) (Figures 3A and S3). Five weeks later, the ability of splenocytes to inhibit mycobacterial growth was assessed using the MGIA (Figure 3A). Time to positivity (TTP), defined as the time taken for mycobacterial growth to reach a threshold, was used as the readout. Higher TTP indicates superior control of mycobacteria.

Splenocytes from BCG-vaccinated animals had significantly improved mycobacterial control compared with those from BCG-unvaccinated mice regardless of the MCMV infection status (Figure 3B, *p* < 0.0001). In splenocytes from unvaccinated mice, MCMV infection significantly improved mycobacterial control compared with MCMV-uninfected mice (Figure 3B, *p* = 0.0029). However, in BCG-vaccinated mice, there was no significant difference in mycobacterial growth control between splenocytes from the MCMV-infected and MCMV-uninfected groups (Figure 3B).

Previous studies in BCG-vaccinated South African infants have shown that CMV infection reduced the BCG- and purified protein derivative (PPD)-specific T cell responses in blood.^13^ This led to the hypothesis that, in CMV-infected infants, both the increased risk of TB disease and the trend toward increased risk of Mtb infection are due to reduced mycobacteria-specific T cell responses. To test whether this was also the case in mice, we determined PPD-specific T cell responses following BCG vaccination and MCMV infection (Figure 3C). However, MCMV did not influence the PPD-specific T cell response in BCG-vaccinated mice (Figure 3C).

**Figure 4:**
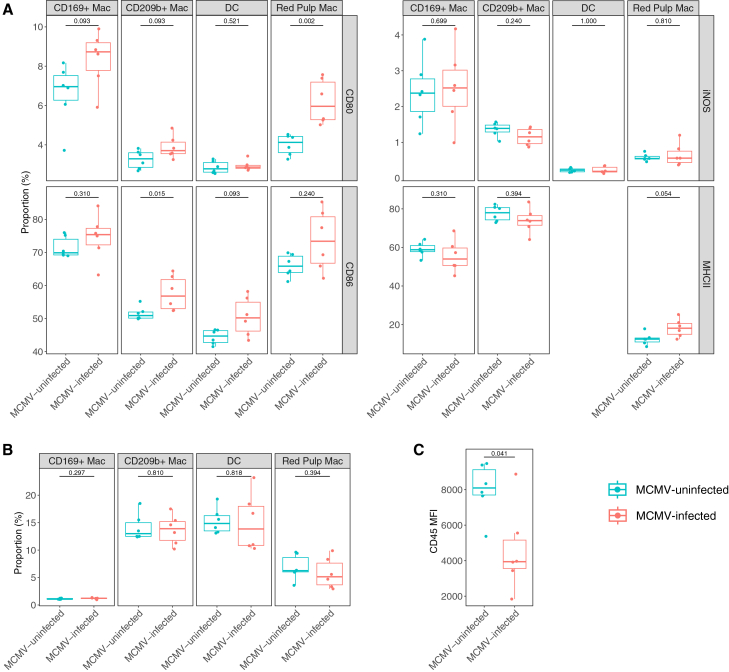
The influence of MCMV infection on the activation marker expression on DCs and macrophages from the spleen (A) The proportion of CD80^+^, CD86^+^, iNOS^+^, and MHC class II^+^ cells among CD169^+^ macrophages (Mac), CD209b^+^ Mac, DCs, and red pulp macrophages of the spleens from MCMV-uninfected (blue) and MCMV-infected (red) mice. (B) The proportion of DCs and macrophage subsets (CD169^+^, CD209b^+^, and red pulp macrophages) within parent populations. Gating strategies are shown in Figure S4. (C) The MFI of CD45 in live Lin^−^ cells from MCMV-uninfected and MCMV-infected mice; Lin, lineage markers, including CD3, CD19, CD49d, and Ly6G. Mann-Whitney test was used to compare the two groups. The boxplot indicates the median value with the interquartile range (IQR). The upper whisker extends to the largest value no further than 1.5× IQR from the hinge, and the lower whisker extends from the hinge to the smallest value at most 1.5× IQR from the hinge. *n* = 6 mice per group. MFI, mean fluorescence intensity. See also Figures S4 and S5.

To investigate how MCMV infection results in the improved mycobacterial growth control by splenocytes in unvaccinated mice, we analyzed the expression of activation markers on dendritic cells (DCs) and macrophages from the spleens of unvaccinated MCMV-infected and -uninfected mice (Figure S4). Macrophages could be divided into three major populations: F4/80^+^CD11b^low^ red pulp macrophages; marginal zone macrophages (MZMs), which highly express C-type lectin SIGN-related 1 (SIGNR1, CD209b); and marginal metallophilic macrophages (MMMs), which highly express sialic acid-binding Ig-like lectin-1 (Siglec-1, CD169).^32^ *In vivo* MCMV infection increased the expression of CD80, CD86, and MHC class II in the macrophage and DC populations in the spleen (Figure 4A). The proportion of CD80^+^ cells was significantly higher in red pulp macrophages from MCMV-infected mice than in those from MCMV-uninfected mice (*p* = 0.002), with a similar trend observed in MMMs (*p* = 0.093) and MZMs (*p* = 0.093), although the difference was not statistically significant (Figure 4A). The proportion of CD86^+^ cells was significantly higher in MZMs from MCMV-infected mice than in those from naive mice (*p* = 0.015), with a similar trend in DCs (Figure 4A, *p* = 0.093). In addition, red pulp macrophages from MCMV-infected mice had a trend toward higher proportion of MHC class II^+^ cells compared with those from MCMV-uninfected mice (Figure 4A, *p* = 0.054). The iNOS expression was similar between MCMV-infected and uninfected mice across all DC and macrophage populations (Figure 4A). Furthermore, the proportions of DCs and different macrophage populations in the spleen were not impacted by MCMV infection (Figure 4B). However, the mean fluorescence intensity (MFI) of CD45 in live Lin^−^ (lineage marker-negative) cells from MCMV-infected mice was significantly lower than in those from uninfected mice (Figure 4C, *p* = 0.041), similar to the results of *in vitro* MCMV infection of macrophages (Figures 1A and S1).

We subsequently compared the phagocytosis rate of BCG-GFP by macrophages and DCs from the spleen of MCMV-infected and MCMV-uninfected mice. In all populations tested, the phagocytosis rate of BCG-GFP was similar between the two groups (Figure S5).

### MCMV infection does not have detrimental effects on the control of mycobacterial growth *in vivo*

**Figure 5:**
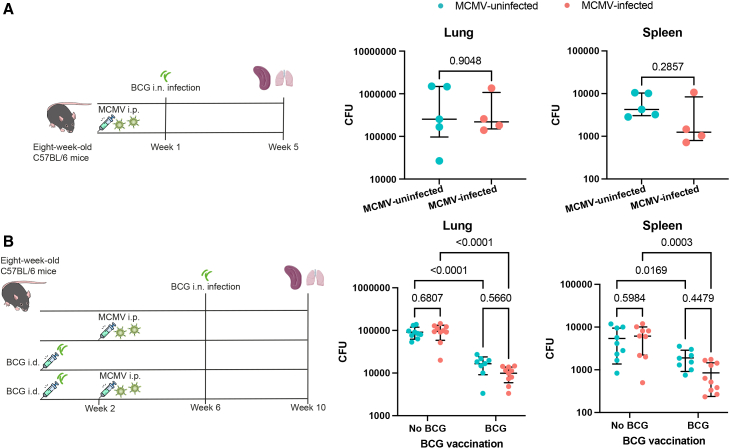
The influence of i.p. MCMV infection on the control of BCG *in vivo* (A) BCG CFUs in the lung and spleen of mice that were infected or uninfected with MCMV. Mann-Whitney test was used to compare the two groups. The error bar indicates the median value with the IQR. The upper whisker extends to the largest value no further than 1.5× IQR from the hinge, and the lower whisker extends from the hinge to the smallest value at most 1.5× IQR from the hinge. *n* = 4–5 mice per group. (B) BCG CFUs in the lung and spleen of BCG-vaccinated or unvaccinated mice that were infected or uninfected with MCMV. Red dots represent mice infected with MCMV, and blue dots represent mice uninfected with MCMV. No BCG means no BCG vaccination of the mice. Uncorrected two-way ANOVA was used to compare the CFUs between different groups. The error bar indicates the mean value with SD. *n* = 8–10 mice per group.

To explore whether MCMV infection influences the ability of mice to control mycobacterial infection, mice were infected with MCMV (i.p.) and subsequently challenged with high-dose intranasal (i.n.) BCG. The lung and spleen bacterial load (in colony-forming units [CFUs]) was measured four weeks post-BCG infection. The one-week interval between MCMV and BCG infection was chosen based on a prospective cohort study that showed an association between early life CMV infection (detected by qPCR of nasopharyngeal swabs)^12^ and increased TB susceptibility later in life. There was no significant difference in the bacterial burden in the spleen or lung between MCMV-infected and -uninfected mice (Figure 5A).

We then extended the interval between MCMV infection and BCG challenge to 4 weeks, at which MCMV infection is considered chronic.^33^^,^^34^^,^^35^ We also introduced two groups of BCG-vaccinated mice to investigate whether MCMV influences the protective efficacy of BCG vaccination (Figure 5B). This extended interval did not impact the ability of MCMV-infected mice to control mycobacteria, and no difference in the bacterial load was observed between BCG-vaccinated MCMV-infected and BCG-vaccinated MCMV-uninfected mice (Figure 5B). However, there was a trend toward lower bacterial burden in the spleen of BCG-vaccinated MCMV-infected mice compared to BCG-vaccinated MCMV-uninfected mice (*p* = 0.0155 when the Mann-Whitney test was used, and *p* = 0.448 when two-way ANOVA was used).

These results suggest that neither acute nor chronic MCMV infection affects BCG growth control in unvaccinated mice. Chronic MCMV infection resulted in a lower CFU in the spleen of BCG-vaccinated mice, but this difference was not statistically significant.

**Figure 6:**
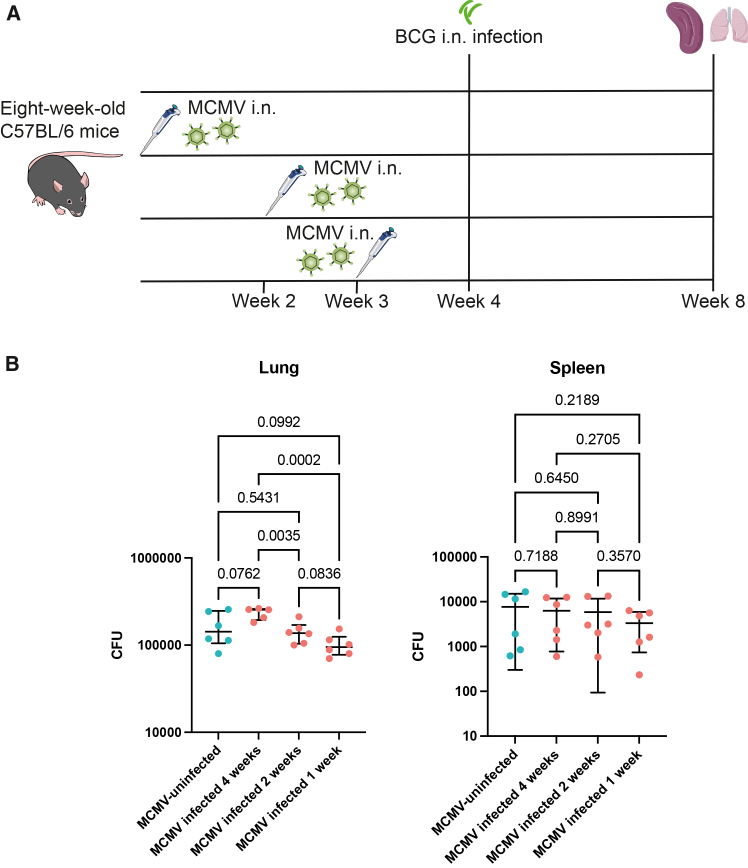
Effect of the interval between i.n. MCMV infection and BCG challenge on mycobacterial control *in vivo* (A) Schematic of the experiment. (B) BCG CFUs in the lung and spleen of BCG-unvaccinated mice that were either uninfected (blue dots) or infected with MCMV (red dots) at 1, 2, or 4 weeks before high-dose BCG infection. The error bar indicates the mean value with SD. Statistical analysis was performed using uncorrected one-way ANOVA. *n* = 5–6 mice per group.

Since breastfeeding was strongly linked to CMV positivity in the nasopharyngeal swab of infants, we changed the route of MCMV infection to i.n. to target the lung mucosal surface.^12^ The i.n. route has been shown to reliably induce a robust infection, in contrast to oral gavage, which results in abortive infection.^33^ To investigate whether the timing of MCMV exposure affects BCG growth control, we varied the interval between i.n. MCMV infection and i.n. BCG challenge (Figure 6A).

There was no significant difference in the bacterial burden in the lung or spleen between MCMV-infected and MCMV-uninfected mice regardless of the interval (Figure 6B). However, as the interval between MCMV infection and BCG challenge shortened, the bacterial burden in the lung of MCMV-infected mice significantly decreased (*p* = 0.0002 for 4-week vs. 1-week interval; *p* = 0.0035 for 4-week vs. 2-week interval), suggesting a non-specific enhancement of mycobacterial control in the lung by MCMV infection.

**Figure 7:**
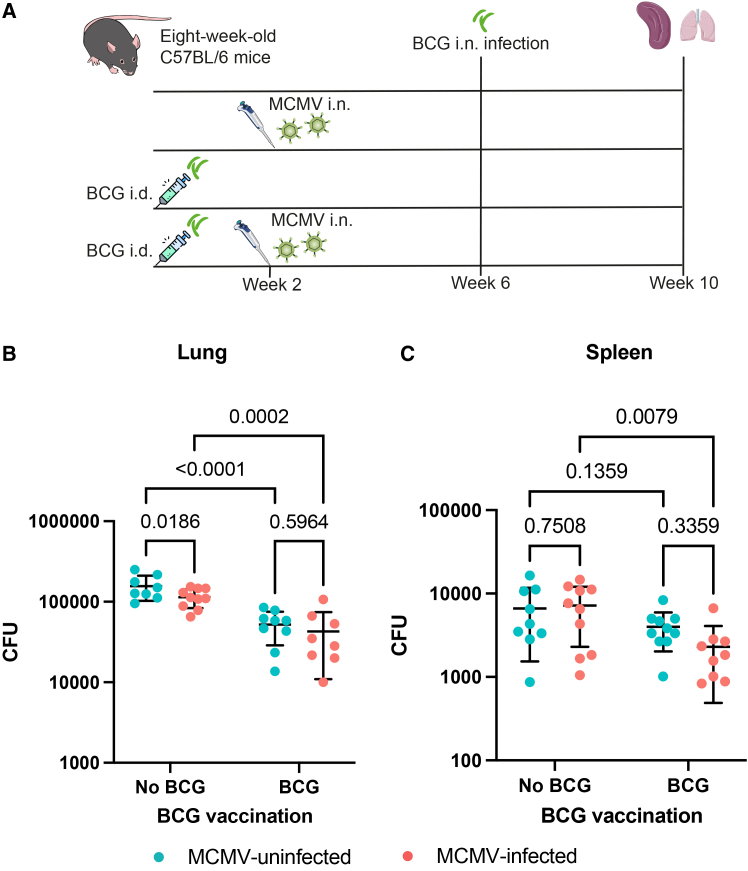
The influence of i.n. MCMV infection on the control of BCG growth *in vivo* (A) Schematic of the experiment. (B and C) CFUs in the lung (B) and spleen (C) of BCG-vaccinated or unvaccinated mice, with or without i.n. MCMV infection. Red dots represent mice infected with MCMV, and blue dots represent mice uninfected with MCMV. Uncorrected two-way ANOVA was used to compare the CFUs between different groups. The error bar indicates the mean value with SD. *n* = 8–10 mice per group.

We next investigated whether MCMV infection influences mycobacterial growth control in BCG-vaccinated mice, using a four-week interval between i.n. MCMV infection and i.n. BCG challenge (Figure 7A). This interval was chosen based on the observation that mice with a four-week interval had a trend for a higher lung bacterial load than those with shorter intervals (Figure 6B). This trend is consistent with findings from human studies.^11^^,^^12^^,^^14^

BCG vaccination significantly reduced the lung bacterial load compared with that in unvaccinated controls, regardless of MCMV infection status (Figure 7B, *p* < 0.0001 in MCMV-uninfected mice vs. *p* = 0.0002 in MCMV-infected mice). In the spleen, however, a protective effect was observed only in MCMV-infected mice (Figure 7C, *p* = 0.0079). Among BCG-vaccinated animals, MCMV infection did not significantly affect the bacterial burden in either the lung or spleen. However, there was a trend toward lower spleen bacterial burden in BCG-vaccinated MCMV-infected mice compared with BCG-vaccinated MCMV-uninfected mice (Figures 7B–7C, *p* = 0.023 when the Mann-Whitney test was used; *p* = 0.336 when uncorrected two-way ANOVA was used).

In unvaccinated mice, MCMV infection was associated with a lower bacterial burden in the lung relative to that in MCMV-uninfected mice (Figure 7B, *p* = 0.0186). However, the effect size was small, and this difference was no longer statistically significant after two-way ANOVA with Tukey’s multiple testing correction (*p* = 0.0823). MCMV infection had no impact on the bacterial load in the spleen (Figure 7C).

## Discussion

In this study, we investigated how prior MCMV infection influences host susceptibility to mycobacterial infection. In the MVA85A efficacy trial of BCG-vaccinated South African infants, CMV infection was associated with an increased risk of TB disease and showed a trend toward an association with increased risk of Mtb infection.^11^^,^^13^ However, whether the association between CMV infection and increased susceptibility to TB was causal or correlative remains unclear. Therefore, the primary aim of this study was to use a murine model of CMV infection to evaluate if CMV infection could causally influence the susceptibility to mycobacterial infection and BCG vaccine immunogenicity. We found that MCMV infection, regardless of previous BCG vaccination, the tested routes of MCMV infection, and the tested intervals between MCMV infection and mycobacterial infection, did not increase the susceptibility to mycobacterial infection *in vivo* in mice. In contrast, both i.p. and i.n. routes of MCMV infection resulted in a trend toward enhancing the control of mycobacteria in the spleen of BCG-vaccinated mice *in vivo*. The discrepancy between the results of this murine study and those from the human study may be due to the use of BCG as a surrogate challenge organism in this study. Unlike Mtb, BCG is less virulent and was used here due to biological safety constraints. Previous studies have shown that infection with an MCMV strain lacking the non-essential m01–m16 genes (eMCMV) (i.v. or i.p. injection) non-specifically reduced the susceptibility of mice to aerosol Mtb infection, particularly during the early stages of infection.^36^ However, it is important to note that this mutant virus lacks several immunomodulatory genes, including MCMV-encoded chemokine 2 (*MCK2*), an important virulent factor necessary for MCMV to infect macrophages.^36^^,^^37^^,^^38^ Another murine herpesvirus, γ-herpesvirus 68 (γHV68), a model for human Epstein-Barr virus (EBV) infection, also improved the ability of mice to control Mtb growth *in vivo.*^39^

It is also important to consider that CMV infection may represent a marker of broader pathogen exposure, rather than acting as a direct causal factor. In the UK, CMV infection in adults was associated with increased exposure to various pathogens, like EBV, Merkel cell polyomavirus, and varicella zoster virus.^40^ This raises the possibility that the observed associations between CMV infection and increased susceptibility to TB could, at least in part, be due to the increased exposure to other pathogens. These complex pathogen-pathogen interactions are difficult to fully recapitulate in specific pathogen-free (SPF) mouse models.

Previous work has shown that the initial MCMV infectious dose can determine the magnitude and quality of heterologous CD8^+^ T cell immunity to LCMV (lymphocytic choriomeningitis virus), with high doses being required to impair responses to subsequent LCMV.^41^ In light of these findings, future studies should assess how variations in MCMV inoculum and the resulting viral replication kinetics (organ-specific viral burden, and persistence and intermittent reactivation) influence downstream mycobacterial T cell responses and susceptibility to mycobacterial infection.

Moreover, the timing of readouts post-challenge might have influenced our findings. For example, in a mouse model, influenza infection increased the bacterial burden only at 120 days post-Mtb infection but not at 63 days or earlier time points.^42^ As the MCMV genome can be detected in the lungs and spleen for up to 26 weeks post-infection, it is possible that differences in susceptibility may be revealed at later time points post-mycobacterial challenge. Another possible explanation for the lack of increased susceptibility is that the mouse strain we used in this study (C57BL/6) is resistant to Mtb infection,^43^ meaning that it can control the infection effectively. As a result, small increases in susceptibility induced by the MCMV could not be detected. Using a more susceptible mouse strain, such as C3H/HeJ, might provide increased sensitivity.

Although the *in vivo* results we generated did not support the hypothesis that MCMV infection influenced the susceptibility to mycobacterial infection, our *in vitro* findings showed that MCMV influences the macrophage phenotype, activation status, mycobacterial phagocytosis, and the ability of macrophage to kill mycobacteria. Previous studies have shown that the gene expression profile of *in vivo* MCMV-infected AMs resembled that of *in vitro* MCMV-infected BMDMs, suggesting that infection with MCMV could override the tissue-specific gene expression profile of macrophages *in vivo*. In contrast, bystander AMs retained a gene expression profile similar to control AMs.^19^ The more pronounced effects of MCMV on macrophages *in vitro* did not translate into a detectable change in susceptibility *in vivo*. This likely reflects the greater complexity of the *in vivo* environment. Even *in vitro*, MCMV had mixed effects on macrophage function: it enhanced BCG killing when autophagy was not induced but impaired autophagy-dependent killing in RAW 264.7 cells, and it did not have any effects on BCG killing in BMDMs and AMs. It also reduced activation and BCG phagocytosis in infected macrophages, while increasing activation in bystander cells. *In vivo*, the added heterogeneity of immune cell populations and the presence of regulatory networks that are absent in monoculture systems likely dilute or mask cell-intrinsic phenotypes.

Previous research has shown that several receptors involved in mycobacterial uptake by macrophages, such as *Marco* (macrophage receptor with collagenous structure), *Mrc1* (mannose receptor, C type 1), *Msr1* (macrophage scavenger receptor 1), and *Cd36*, were downregulated at the gene or protein level in MCMV-infected BMDMs compared with uninfected controls.^19^^,^^44^ The same study reported reduced gene expression of *Marco* and *Mrc1* in bystander BMDMs relative to uninfected controls,^19^ providing a potential explanation for the reduced phagocytosis of BCG-GFP by both MCMV-infected and bystander macrophages in our study. Impaired phagocytosis was also observed with other pathogens; MCMV-infected AMs failed to uptake *Lp* and control its growth *in vivo.*^19^ Similar patterns were also described in human monocytes infected with HCMV, where the gene expression of *MRC1,* *CR3* (complement receptor 3) and *CD36*, as well as the protein level of CD36 were downregulated relative to those in uninfected monocytes,^45^ which was associated with reduced phagocytosis of fungal pathogens.^45^ However, we did not directly measure receptor expression under the specific experimental conditions used in the present study. Therefore, while receptor downregulation represents a plausible mechanism consistent with prior literature, its contribution to the reduced phagocytosis observed here remains to be formally validated in future experiments.

We also observed differences in activation markers between the infected and bystander macrophages. Previous studies have shown that genes and proteins related to interferon responses were upregulated in bystander BMDMs but downregulated in MCMV-infected BMDMs compared with those in uninfected BMDMs.^19^ MCMV-infected macrophages had reduced expression of activation markers, whereas bystander macrophages had increased expression of these markers, potentially compensating for the suppressed phenotype of their infected counterparts. In RAW 264.7 cells, increased expression of iNOS, a molecule essential for controlling mycobacterial growth,^26^ in bystander cells was associated with enhanced killing of mycobacteria. This association was not observed in BMDMs, where the expression of iNOS was increased in bystander cells compared with control cells. AMs showed a more regulatory phenotype, with reduced expression of iNOS in bystander AMs compared with those in bystander BMDMs and RAW 264.7 cells. Interestingly, splenocytes from MCMV-infected mice better controlled BCG growth in the MGIA compared with splenocytes from uninfected mice, despite the lack of iNOS expression in splenic macrophages or DCs. These results suggest that mycobacterial control *in vitro* cannot be simply attributed to iNOS expression in macrophages.

The enhanced control of BCG growth by splenocytes in MCMV-infected mice compared with those from MCMV-uninfected mice was observed only in BCG-unvaccinated mice, suggesting that previous MCMV infection “non-specifically” improved mycobacterial control in unvaccinated mice, whereas BCG vaccination may simply override this effect. It is also possible that the MGIA was not sensitive enough to detect differences in BCG growth between MCMV-infected and uninfected BCG-vaccinated mice or that MCMV infection had minimal impact on mycobacterial control in this context.

Autophagy was also shown to be differentially affected. In MCMV-uninfected RAW 264.7 cells, starvation-induced autophagy resulted in better killing of BCG. However, autophagy did not affect the control of BCG in MCMV-treated RAW 264.7 cells. Previous work showed that MCMV-infected BMDMs had reduced expression of autophagy-related genes, such as *Atg7*, *Atg5*, and *Atg16L2*, compared with uninfected BMDMs,^19^ suggesting that autophagy-mediated mycobacterial killing might be impaired. In AMs, neither autophagy nor MCMV influenced the control of BCG. *In vivo*, autophagy in macrophages can be induced by IFN-γ, suggesting that ongoing MCMV infection of macrophages might impair this by interfering with T cell-mediated macrophage activation. Despite this, the mice infected with i.n. MCMV one week before i.n. BCG infection had lower lung bacterial burden than the mice with a 4-week interval between MCMV infection and BCG infection, suggesting that the non-specific activation of the immune system by acute MCMV infection dominated over its detrimental effects on the immune system.

In the MVA85A efficacy trial, CMV-infected infants had reduced PPD- and BCG-specific T cell responses compared with CMV-uninfected infants.^13^ Similarly in mice, MCMV infection decreased pre-existing memory T cells induced by Ad-LacZ immunisation.^46^ This was shown to be through the bystander activation of the Fas-dependent apoptotic pathway.^46^ Studies have also demonstrated that MCMV can downregulate co-stimulatory molecules such as CD80 and upregulate co-inhibitory molecules such as PD-L1 (program death ligand 1) in DCs, which can further lead to reduced T cell proliferation, effector function, and recall response of T cells against irrelevant antigens.^47^^,^^48^ This leads to the hypothesis that acute MCMV infection might reduce the T cell response to BCG vaccination through similar mechanisms. In the MVA85A efficacy trial, infants were found to be infected with CMV at 4–6 months post-BCG vaccination, which coincided with the peak of PPD-specific T cell response in South African infants.^49^ In the murine study, the interval between MCMV infection and BCG vaccination was selected to be two weeks, as this corresponded to the peak of the PPD-specific T cell response observed at two weeks post-BCG vaccination in mice.^50^ However, MCMV infection did not influence the PPD-specific T cell response of splenocytes from BCG-vaccinated mice. Further experiments with varying intervals between MCMV infection and BCG vaccination and focusing on the impact of different BCG doses may be necessary to fully understand how MCMV influences the T cell response to BCG. The relatively high BCG dose used, equivalent to the standard human dose, may have masked more subtle effects of prior MCMV infection, and it is possible that lower doses could yield different outcomes.

In summary, this study demonstrated that MCMV infection did not negatively impact the control of mycobacteria in mice. However, MCMV infection did have an impact on the phenotype, activation, phagocytosis, and *in vitro* mycobacterial killing in macrophages *in vitro*. To further investigate the potential effects of MCMV on mycobacterial control, future studies could benefit from the utilization of Mtb (including hypervirulent strains of Mtb) and mouse strains that are more susceptible to Mtb. These changes may increase the sensitivity of the model and allow the detection of potential differences between MCMV-infected and MCMV-uninfected mice.

### Limitations of the study

This study has several limitations. First, the mouse strain used is relatively resistant to both MCMV and mycobacterial infections compared with other commonly used strains, such as BALB/c and C3H/HeJ. Second, we employed BCG, a live attenuated strain of *Mycobacterium bovis*, rather than a virulent Mtb strain, which may limit the relevance of our findings to natural Mtb infection. In addition, we tested only a fixed dose of BCG vaccination and MCMV infection and examined a limited number of intervals between BCG vaccination and MCMV infection, as well as between MCMV infection and mycobacterial challenge. More comprehensive studies using alternative mouse and mycobacterial strains, as well as varied dosing regimens and timing of infections are required to extend our findings and better define the interactions between CMV and Mtb.

Our study also shares some technical limitations common to the MCMV field. First, our viral stocks were produced in NIH 3T3 cells (NIH/Swiss background); so host cell-derived proteins in these preparations could, in principle, elicit alloreactive responses when used to infect C57BL/6 mice. In future work, generating MCMV stocks on primary mouse embryonic fibroblasts (MEFs) from the same mouse strain used *in vivo* (e.g., C57BL/6) would minimize this concern. Second, we used the RAW 264.7 macrophage cell line, which has been reported to harbor ecotropic and polytropic murine leukemia viruses, potentially influencing baseline activation or antiviral pathways.^51^ Although both NIH 3T3-derived stocks and RAW 264.7 cells are standard tools in the field, future studies using MEF-derived virus stocks will be important to confirm and extend our findings.

Another potential limitation of our *in vitro* experiments is the use of crude MCMV preparations, in which only cellular debris was removed by low-speed centrifugation. As a result, the viral inoculum may contain host-derived soluble factors released from infected NIH3T3 cells, including cytokines, interferons, and damage-associated molecular patterns (DAMPs), which could influence the macrophage activation states. Although MCMV-treated cells were washed with PBS 2 h post-infection, we cannot fully exclude the contribution of residual soluble mediators. Therefore, comparisons between macrophages cultured in medium alone (uninfected) and those exposed to virus-containing supernatant (bystander and infected) reflect combined effects of viral infection and any remaining infection-associated soluble factors.

In the present study, infected and bystander macrophages were distinguished based on infection-associated downregulation of CD45 (RAW264.7 cells and BMDMs) or CD11c/SiglecF (AMs), as previously established using purified MCMV.^19^^,^^21^ Because we used crude virus preparations, it was important to confirm that this gating strategy remained valid under these conditions. Our independent validation experiment using MCMV-GFP demonstrates a concordance between GFP expression and loss of CD45, confirming that marker downregulation reliably identifies infected cells, even when crude viral stocks are used. These findings are in agreement with those of previously published studies performed using purified MCMV.^21^

## Resource availability

### Lead contact

Further information or requests should be directed to and will be fulfilled by the lead contact, Elena Stylianou (elena.stylianou@ndm.ox.ac.uk).

### Materials availability

This study did not generate new unique reagents. All materials used in this study are commercially available or available upon reasonable request.

### Data and code availability

• All data and metadata associated with this study are available in the main text and its supplemental information.
• This study did not generate any original code.
• Additional information needed to reanalyze the data presented in this paper can be obtained from the lead contact upon request.

## Acknowledgments

We thank Laura Thomas, Angela Curran, and James Bussell for all the support and advice with the *in vivo* work; Marco Polo Peralta Alvarez and Salem Almujri for their useful advice on the macrophage phagocytosis assay; Catherine de Lara, Narayan Ramamurthy, and Philipp Hackstein for their suggestions on the preparation and quantification of MCMV stocks; and Xiawei Zhang for the support on the isolation and culture of BMDMs and AMs. S.L. would like to acknowledge the China Scholarship Council (CSC) – Nuffield Department of Medicine (NDM) award for providing support for the DPhil study at University of Oxford, UK. This research was supported by the 10.13039/100010269Wellcome Trust. H.M. is a Wellcome Trust Investigator (grant code WT 206331/Z/17/Z). For the purpose of open access, the author has applied a CC BY public copyright license to any “author accepted manuscript” version arising from this submission.

## Author contributions

Conceptualization, S.L., E.S., and H.M.; investigation, S.L., C.H., M.K., C.D.V., A.A., and E.S.; methodology, S.L., C.H., M.K., C.D.V., A.A., P.K., I.S., and E.S.; funding acquisition, H.M.; resources, P.K., E.S., and H.M.; supervision, E.S. and H.M.; writing – original draft, S.L.; writing – review & editing, S.L., C.H., M.K., C.D.V., A.A., P.K., I.S., E.S., and H.M. All authors have reviewed and approved the final manuscript.

## Declaration of interests

The authors declare no competing interests.

## STAR★Methods

### Key resources table

**Table:** 

REAGENT or RESOURCE	SOURCE	IDENTIFIER
**Antibodies**		

PE/Cyanine5 anti-mouse CD64 (FcγRI) Antibody	BioLegend	Cat#139331; RRID: AB_2922467
PE anti-mouse CD11c Antibody	BioLegend	Cat#117307; RRID: AB_313776
APC/Cyanine7 anti-mouse CD170 (Siglec-F) Antibody	BioLegend	Cat#155531; RRID: AB_2904295
Brilliant Violet 605™ anti-mouse CD45 Antibody	BioLegend	Cat#103155; RRID: AB_2650656
MHC Class II (I-A/I-E) Monoclonal Antibody (M5/114.15.2), Alexa Fluor™ 700, eBioscience™	Thermo Fisher	Cat#56-5321-82; RRID: AB_494009
Brilliant Violet 421™ anti-mouse CD86 Antibody	BioLegend	Cat#105123; RRID: AB_2892270
iNOS Antibody, anti-mouse, REAfinity™	Miltenyi Biotec	Cat#130-116-359; RRID: AB_2727485
Brilliant Violet 650™ anti-mouse CD80 Antibody	BioLegend	Cat#104731; RRID: AB_11147759
Brilliant Violet 785™ anti-mouse/human CD11b Antibody	BioLegend	Cat#101243; RRID: AB_2561373
PE/Cyanine7 anti-mouse F4/80 Antibody	BioLegend	Cat#123113; RRID: AB_893490
Brilliant Violet 510™ anti-mouse CD3ε Antibody	BioLegend	Cat#100353; RRID: AB_2565879
Brilliant Violet 510™ anti-mouse CD19 Antibody	BioLegend	Cat#115545; RRID: AB_2562136
BD OptiBuild™ BV510 Hamster Anti-Mouse CD49b	BD Biosciences	Cat#740133; RRID: AB_2739890
Brilliant Violet 510™ anti-mouse Ly-6G Antibody	BioLegend	Cat#127633; RRID: AB_2562937
APC/Fire™ 750 anti-mouse CD11c Antibody	BioLegend	Cat#117351; RRID: AB_2572123
PE/Dazzle™ 594 anti-mouse CD169 (Siglec-1) Antibody	BioLegend	Cat#142423; RRID: AB_2750058
CD209b (SIGN-R1) Antibody, anti-mouse, REAfinity™	Miltenyi Biotec	Cat#130-117-786; RRID: AB_2727966

**Bacterial and virus strains**		

Murid betaherpesvirus 1 strain Smith (MCMV Strain Smith)	ATCC	Cat#VR-1399
*Mycobacterium tuberculosis* variant *bovis* BCG strain TMC 1011 (BCG Pasteur)	ATCC	Cat#35734
BCG-GFP	Professor Rajko Reljic of St. George’s University of London	N/A

**Biological samples**		

Fetal calf serum	Sigma	Cat#F9665

**Chemicals, peptides, and recombinant proteins**		

RPMI 1640	Sigma	Cat#R0883
L-glutamine	Gibco	Cat#G7513
Penicillin-Streptomycin: 10,000u/ml penicillin and 10,000 μg/mL streptomycin sulfate	Gibco	Cat#P0781
Dimethyl sulfoxide	Sigma	Cat#D2650
RPMI 1640 with HEPES	Sigma	Cat#R5886
MEM medium	Sigma	Cat#M4526
DMEM medium	Sigma	Cat#D6546
Bovine serum albumin (for flow cytometry)	Sigma	Cat#A7906
PPD (tuberculin purified protein derivative)	Staten Serum Institute	Cat#2391
M38 peptide	ProImmune Ltd	–
M45 peptide	ProImmune Ltd	–
TrypLE^TM^ Express Enzyme (no phenol red)	Thermo Fisher	Cat#12604013
LIVE/DEAD™ Fixable Red Dead Cell Stain Kit	Thermo Fisher	Cat#L23102
LIVE/DEAD™ Fixable Aqua Dead Cell Stain Kit	Thermo Fisher	Cat#L34957
Anti-Mo CD16/CD32	Invitrogen	Cat#14-0161-85
BD Cytofix/Cytoperm™ Plus Fixation/Permeabilization Solution Kit with BD GolgiPlug™	BD Biosciences	Cat#555028
Dulbecco′s Phosphate Buffered Saline	Sigma	Cat#D8537
ELISpot plates (MultiScreen-IP)	Merck Millipore	Cat#MAIPS4510
Anti-mouse IFN-γ mAb (AN18)	Mabtech	Cat#3321-3-1000
Streptavidin-ALP	Mabtech	Cat#3310-8-1000
AP Conjugate Substrate Kit	BioRad	Cat#1706432
PBS with 0.05% Tween 20	Sigma	Cat#P3563
Water	Sigma	Cat#W3500
Methyl cellulose	Sigma	Cat#274429
MEM (Temin’s modification) (2X), no phenol red (for quantification of MCMV)	Thermo Fisher	Cat#11935046
Crystal violet	Sigma	Cat#C0775
BD DIFCO™ Tween™ 80 Polysorbate 80 100g	Becton Dickinson	Cat#231181
BD™BD BBL™ Middlebrook OADC 6x100 mL	Becton Dickinson	Cat#212240
Glycerol	Sigma	Cat#49767
Tyloxapol	Sigma	Cat#T8761
Middlebrook ADC Enrichment	Becton Dickinson	Cat#212352
BD DIFCO™ Middlebrook 7H9 Broth 500g	Becton Dickinson	Cat#271310
BD Difco™ Middlebrook 7H10 Agar	Becton Dickinson	Cat#262710
Recombinant Murine M-CSF	Peprotech	Cat#315-02
UltraPure™ 0.5M EDTA, pH 8.0	Thermo Fisher	Cat#15575020
Recombinant Murine GM-CSF	Peprotech	Cat#315-03
Earle′s Balanced Salts	Sigma	Cat#E6267
OneComp eBeads	Thermo Fisher	Cat#01-1111

**Critical commercial assays**		

BD BACTEC™ MGIT™ Barcoded 7 mL Tube	Becton Dickinson	Cat#245122
BD BACTEC™ MGIT™ 960 Supplement Kit (100 Tests)	Becton Dickinson	Cat#245124

**Experimental models: Cell lines**		

NIH 3T3 cells	ATCC	Cat#CRL-1658
RAW 264.7 cells	ATCC	Cat#TIB-71

**Experimental models: Organisms/strains**		

Mouse: C57BL/6JOlaHsd	Envigo, UK	Cat#057

**Software and algorithms**		

Prism 10.0.3	GraphPad Software	https://www.graphpad.com/
R v 4.1.2	R Core Team, 2013	https://www.r-project.org/
FlowJo v10.8.1	FlowJo, LLC	https://www.flowjo.com/

### Experimental model and study participant details

#### Animals

Six-week-old female C57BL/6 mice were purchased from Envigo, UK. Animals were group housed in individually ventilated cage (IVCs) under specific pathogen-free (SPF) conditions, with constant temperature and humidity. Housing conditions were as follows: temperature: 22°C–24°C, 12 h day/night cycle, humidity 40–70%. Sex differences were not assessed in this study. All animal work was approved by the University of Oxford Animal Welfare and Ethical Review Board and subsequently approved by the UK Home Office under project licenses PPL PP3430109, 30/2889, and P9804B4F1, in accordance with the UK Animals (Scientific Procedures) Act 1986. All procedures were carried out by appropriately trained and licensed personnel.

#### RAW 264.7 cells

RAW 264.7 cells (ATCC TIB-71, from an adult male BALB/c mouse) were cultured in the D10 medium (See Table S1 for the formulation of all medium and buffers used in this study) at 37 °C, 5% CO_2_. A T75 flask was seeded with 1-3×10^6^ cells and subcultured every 3–5 days when the cells reached 70–80% confluence. Cells were detached using the Tryp^LE^ Express enzyme and a cell scraper and resuspended in the D10 medium.

#### NIH3T3cells

NIH 3T3 cells (ATCC CRL-1658, from a male Swiss mouse embryo) were cultured in the D10 medium at 37 °C, 5% CO_2_. A T75 flask was seeded with 1×10^6^ cells and subcultured every 2–3 days when the cells reached 70–80% confluence. Cells were detached using Tryp^LE^ Express enzyme and resuspended in the D10 medium.

#### Bone marrow-derived macrophages (BMDMs)

Adult female C57BL/6 mice were sacrificed, and femur and tibia were removed and placed in room temperature PBS. After cleaning and joint removal, the marrow was flushed using PBS in a syringe with a 25G needle. The marrow was gently pipetted and passed through a cell strainer into a collection tube. The cells were centrifuged at 250×g for 5 min at room temperature, the supernatant was discarded, and the pellet was resuspended in the macrophage growth medium. This suspension was distributed across approximately 15 petri dishes per mouse.

During macrophage culturing, an additional 5 mL macrophage growth medium was added on day 3 or 4. For harvesting differentiated BMDMs on day 7, the macrophage culture medium was removed, and cells were rinsed with 5 mL of PBS at room temperature and then 5 mL of cold PBS containing 5 mM EDTA for 3 min. Cells were dislodged gently by pipetting using a plastic transfer pipette. After washing again with 5 mL of cold PBS containing 5 mM EDTA, the cells were collected by centrifugation at 250×g for 5 min at room temperature.

Cells were counted using a hemocytometer, and a sample was, in some instances, collected for flow cytometry analysis to assess culture purity. The cells were then resuspended in macrophage growth medium and plated in 12-well tissue culture plates at a density of 2×10^5^ cells per well.

#### Alveolar macrophages

Adult female C57BL/6 mice were euthanized, and the trachea was exposed by opening the thorax. A 27G catheter was then inserted into the trachea. PBS containing 2 mM EDTA was infused to inflate the lungs, and the resulting cell mixture was withdrawn. Following centrifugation, the cell pellet was placed into 24-well plates in the R10 medium supplemented with 20 ng/mL of GM-CSF. After allowing cells to adhere for 2 h, non-adherent cells were washed away, leaving behind adherent macrophages ready for infection.

#### Preparation of MCMV (or MCMV-GFP) stock

NIH 3T3 cells were seeded in a T175 flask to reach 30–40% confluency by the following day. Cells were then washed with PBS and infected with MCMV (Strain Smith, ATCC VR-1399, MOI 2–3) or MCMV-GFP (MOI 2–3) in D2 medium at 37 °C, 5% CO_2_ for 2 h with occasional rocking. After adding 5 mL more medium, cultures were incubated at 37 °C, 5% CO_2_ for 3–4 days until cytopathic effects were observed. Supernatants were collected by centrifuging at 1,500×g for 10 min, aliquoted, and stored at −80 °C.

#### Preparation and quantification of BCG (or BCG- GFP) stock

BCG Pasteur was grown in Middlebrook’s 7H9 broth supplemented with 0.05% Tween-80 and 10% Albumin Dextrose Catalase (ADC) at 37 °C under aerobic conditions with continuous shaking at 200 rpm. The BCG Montreal strain (ATCC 35735) which harbors the pEGFP plasmid under the regulation of the mycobacterial 19 kDa gene promoter, was generously provided by Professor Rajko Reljic of St. George’s University of London.^52^ This strain, referred to as BCG-GFP, was cultivated in Middlebrook’s 7H9 broth supplemented with 10% Oleic Albumin Dextrose Catalase (OADC), 0.2% glycerol and 0.05% tyloxapol, and the culture was maintained at 37 °C under aerobic conditions with continuous shaking at 200 rpm. BCG was quantified by plating in serial dilutions on Middlebrook 7H10 agar plates supplemented with 10% OADC and 0.5% glycerol. Quantification of BCG-GFP was performed by measuring the optical density at 600 nm (OD600) using a spectrophotometer.

### Method details

#### Quantification of MCMV (or MCMV-GFP)

NIH 3T3 cells were seeded and incubated overnight before infection with serial dilutions of MCMV (or MCMV-GFP) in the D2 medium. After a 2-h incubation at 37 °C, the inoculum was removed and replaced with a 1:1 CMC/MEM (plaque assay). Plates were incubated for 3–4 days, then fixed and stained with 0.1% crystal violet in 20% ethanol. The crystal violet was then aspirated, and the plates were left to dry. Plaques were counted to determine virus titers.

#### *In vitro* infection of MCMV

Macrophages were seeded in 12-well plates at a density of 2×10^5^ cells per well in 1 mL of the D10 medium (20 ng/mL M-CSF and 20 ng/mL GM-CSF were added for BMDMs and AMs, respectively. R10 medium was used for AMs). Cells were incubated overnight. The next day, the cells were infected with MCMV (or MCMV-GFP) at an MOI of 1–10 in the MCMV infection medium (RAW 264.7 cells: MOI = 10; BMDMs: MOI = 2; AMs: MOI = 1). Cells were centrifuged at 400×g for 30 min at room temperature to enhance infection and then incubated at 37 °C for 2 h. After the incubation period, the cells were washed with 1 mL of PBS, and 1 mL of fresh MCMV infection medium was added. Two days post-infection, the cells were further processed for flow cytometry, BCG-GFP phagocytosis assay or BCG killing assay.

#### *In vitro* BCG-GFP phagocytosis assay

BCG-GFP was harvested at the log phase and washed once with 0.05% PBS-Tween80 (3,000×g, 10 min). The pellet was resuspended in 10 mL of 0.05% PBS-Tween80, supplemented with 10–15 sterile glass beads, and vortexed for 1 min. Eight mL of the suspension was then transferred to a 50 mL tube and passed through a 26G needle three times to break up clumps. The suspension was then sonicated 10 times for 30 s each, with 30 s rest in between, and then was measured for OD600 to determine the concentration. An OD600 of 0.1 was equivalent to 1 × 10^7^ CFU/mL. Cells were washed with PBS, and MCMV infection medium (no antibiotics) was added. BCG-GFP was added to the cells at an MOI of 10 for 4 h at 37 °C. Cells were processed for flow cytometry to measure phagocytosis rate. For BCG-GFP phagocytosis assay using splenocytes, BCG-GFP with 1×10^6^ CFU was added to 1×10^6^ splenocytes resuspended in 100 μL of the M10 medium (no antibiotics) and incubated for 4 h at 37 °C.

#### *In vitro* BCG killing assay

The assay was set up according to previous studies.^29^^,^^53^^,^^54^ Frozen BCG was thawed at room temperature and sonicated three times for 60 s each, with 60 s rest in between. Cells were washed with PBS and resuspended in the macrophage growth medium without antibiotics. BCG was then added to cells at an MOI of 1. Macrophages were then centrifuged at 500×g for 5 min at room temperature to settle BCG onto macrophages. The cells were then incubated at 37 °C for 1 h and then washed twice with macrophage growth medium without antibiotics but with 50 μg/mL gentamycin to kill extracellular BCG. The cells were then divided into three groups: normalization group, in which cells were harvested immediately after the 1-h incubation; full nutrient group, in which cells were incubated for another 2 h in 500 μL of the macrophage growth medium without penicillin-streptomycin but with gentamycin at 50 μg/mL; and nutrient-deprived group, in which cells were incubated for another 2 h in the EBSS with 50 μg/mL gentamycin to induce autophagy.

To harvest intracellular BCG from the full nutrient and nutrient-deprived groups, 500 μL of the medium was collected and centrifuged at 13,000×g for 10 min. 500 μL of cold deionized water was then added. The mixture was incubated at 4 °C for 10-15 min to lyse the cells and release intracellular BCG. After centrifugation of the 500 μL medium, 400 μL supernatant was removed. The rest of the pellet and supernatant was combined with the 500 μL lysate. To harvest intracellular BCG from the control group, 600 μL of cold deionized water was added and incubated for 10–15 min. The 600 μL liquid containing BCG was then transferred to a BACTEC MGIT tube supplemented with BBL MGIT OADC and PANTA. MGIT tubes were placed on the BACTEC 960 machine (Becton Dickinson, UK) and incubated at 37°C until the detection of positivity by fluorescence.

TTP readout was converted to CFU using standard curves. CFU from the full nutrient group and nutrient-deprived group were then normalized to the normalization group to remove the difference in the number of intracellular BCG between MCMV-treated cells and MCMV-untreated cells due to different phagocytosis rates.

#### *In vivo* infection of MCMV and BCG

Six-week-old female C57BL/6 mice were purchased from Envigo, UK. Animals were group housed in IVCs under SPF conditions, with constant temperature and humidity. For intraperitoneal infection of MCMV, 8- to 10-week-old mice were injected with 2 × 10^6^ PFU of MCMV in 50 μL of the D2 medium. For intranasal infection of MCMV, 8- to 10-week-old mice were anesthetized with isoflurane and injected with 4 × 10^5^ PFU of MCMV in 30 μL of the D2 medium. For intranasal infection of BCG, 9- to 14-week-old mice were anesthetized with isoflurane and administered with 5 × 10^6^ CFU of BCG in 30 μL of the 7H9 media. Four weeks after the intranasal infection of BCG, mouse lungs and spleens were harvested and homogenized in 1 mL PBS in Precellys tubes before plating on 7H10 agar plates in serial dilutions for CFU determination of BCG. For intradermal vaccination of BCG, 8-week-old mice were injected with 3.5 × 10^5^ CFU of BCG in 50 μL of PBS.

#### Organ processing

Mice were euthanized by cervical dislocation, and the spleen was removed and placed in 1 mL of RPMI 1640 medium. Spleens were mashed through a 70 μm cell strainer in PBS and spun at 500×g for 5 min. Red blood cells were then lysed using Ammonium-Chloride-Potassium (ACK) lysis buffer. Cells were resuspended in 2 mL of the R10 medium (HEPES, no antibiotics) for MGIA assay and 2 mL of the M10 medium for ELISpot assay and flow cytometry. Cells were then counted and resuspended in the appropriate medium for downstream assays. The remaining splenocytes were spun down and resuspended in 1 mL of FCS with 10% DMSO for cryopreservation.

#### Flow cytometry

##### Macrophage immune phenotyping

Approximately 2×10^5^ macrophages were harvested using Tryp^LE^ Express enzyme for 20 min at 37 °C, washed with PBS, and stained with fixable live/dead dye for 10 min at 4 °C. Cells were then incubated with Fc block, followed by surface staining with an antibody cocktail for 30 min at 4 °C. After washing, cells were fixed, permeabilised, and stained intracellularly with antibodies for 30 min at 4 °C. Following a final wash, cells were resuspended in 2% BSA/PBS for acquisition on a BD LSRFortessa (BD Biosciences). Antibodies used for macrophage immune phenotyping are listed in key resources table.

##### Immune phenotyping of macrophages and DCs using frozen splenocytes

Frozen splenocytes were rapidly thawed at 37 °C in a water bath. Thawed splenocytes were gradually added to 9 mL of warm M10 medium. The cells were then centrifuged at 500×g for 7 min at room temperature. After centrifugation, the supernatant was discarded, and the cell pellet was resuspended in 2 mL of the M10 medium. Cells were counted and resuspended in M10 medium at a concentration of 1×10^7^ cells/ml. 100 μL of the cell suspension was transferred to a 96-well U-bottom plate and was incubated in 5% CO_2_ at 37 °C for 4 h. Cells were washed with PBS and stained as described in the *in vitro* macrophage immune phenotyping section.

#### Enzyme-linked immunoSpot (ELISpot) assay

Fresh splenocytes were resuspended in the M10 medium at a concentration of 1×10^7^/mL. Plates were coated with an anti-mouse-IFN-γ capture antibody and then blocked with M10 medium. Post blocking, 5×10^5^, 2.5×10^5^ or 1.25×10^5^ splenocytes in 50 μL of the M10 medium were added in the wells. Cells were stimulated in triplicate wells with 10 μg/mL of PPD-T, 2 μg/mL of the M38 peptide (SSPPMFRV), 2 μg/mL of the M45 peptide (HGIRNASFI), or left unstimulated as a negative control for 16–18 h. Plates were washed again with PBS/0.05% Tween 20, then incubated of a biotinylated anti-mouse-IFN-γ antibody, followed by incubation with streptavidin-ALP. Post-wash plates were developed with developer and stopped by washing with tap water. Plates were dried overnight and were read the next day. Spots were counted using an ELISpot reader system ELR02 (AID Diagnostika, v7.0 iSpot).

#### Mycobacterial growth inhibition assay (MGIA)

Spleens of mice were collected and processed as described in the murine organ processing section. Five million splenocytes were incubated with BCG Pasteur diluted at 1/7000 (270 CFU) FCS in a total volume of 600 μL/well in a 48-well plate. Cultures were incubated at 37 °C for 96 h. Extracellular and lysed intracellular BCG were transferred to the BACTEC MGIT tubes supplemented with BBL MGIT OADC and PANTA. Tubes were placed on the BACTEC 960 machine and incubated at 37 °C until the detection of positivity by fluorescence. TTP was used as a readout in this study. Control tubes were set up by inoculating supplemented BACTEC MGIT tubes with the same volume of BCG as the samples.

### Quantification and statistical analysis

Flow cytometry data were acquired on BD Fortessa Flow Cytometer (BD Biosciences) and processed in FlowJo version 10.8.1 (FlowJo, LLC). Spots in the ELISpot assay were counted using an ELISpot reader system ELR02 (AID Diagnostika, v7.0 iSpot). Statistical analysis was performed with GraphPad Prism (GraphPad software, Version 10.0.3) and R 4.1.2. For comparison of two groups, unpaired two-tailed Mann-Whitney test was performed. For multiple comparisons, one- or two-way ANOVA were performed, followed by Tukey’s multiple comparison tests for all *in vitro* and *ex vivo* experiments or left uncorrected for the *in vivo* experiments, as stated in the figure legend. *p* values were reported in each figure. Data are presented as mean ± SD or median ±1.5 × IQR as stated in the figure legend. The number of mice or biological samples to generate the data and other statistical details of experiments are shown in the figure legend. Sample exclusion was performed for the CFU data when the agar plates used for CFU counting were contaminated.
