# Supplementary material for: The influence of murine cytomegalovirus infection on susceptibility to mycobacterial infection
Source: iScience. 2026 Mar 27;29(5):115518. doi: 10.1016/j.isci.2026.115518 (PMC13098500; doi:10.1016/j.isci.2026.115518)
Supplement: Document S1. Figures S1–S5 and Table S1 [file mmc1.pdf]

iScience, Volume *29*

Supplemental information

The influence of murine cytomegalovirus infection on susceptibility to mycobacterial infection

Shuailin Li, Claire Hutchings, Marcellus Korompis, Christopher De Voss, Alberta Ateere, Iman Satti, Paul Klenerman, Helen McShane, and Elena Stylianou

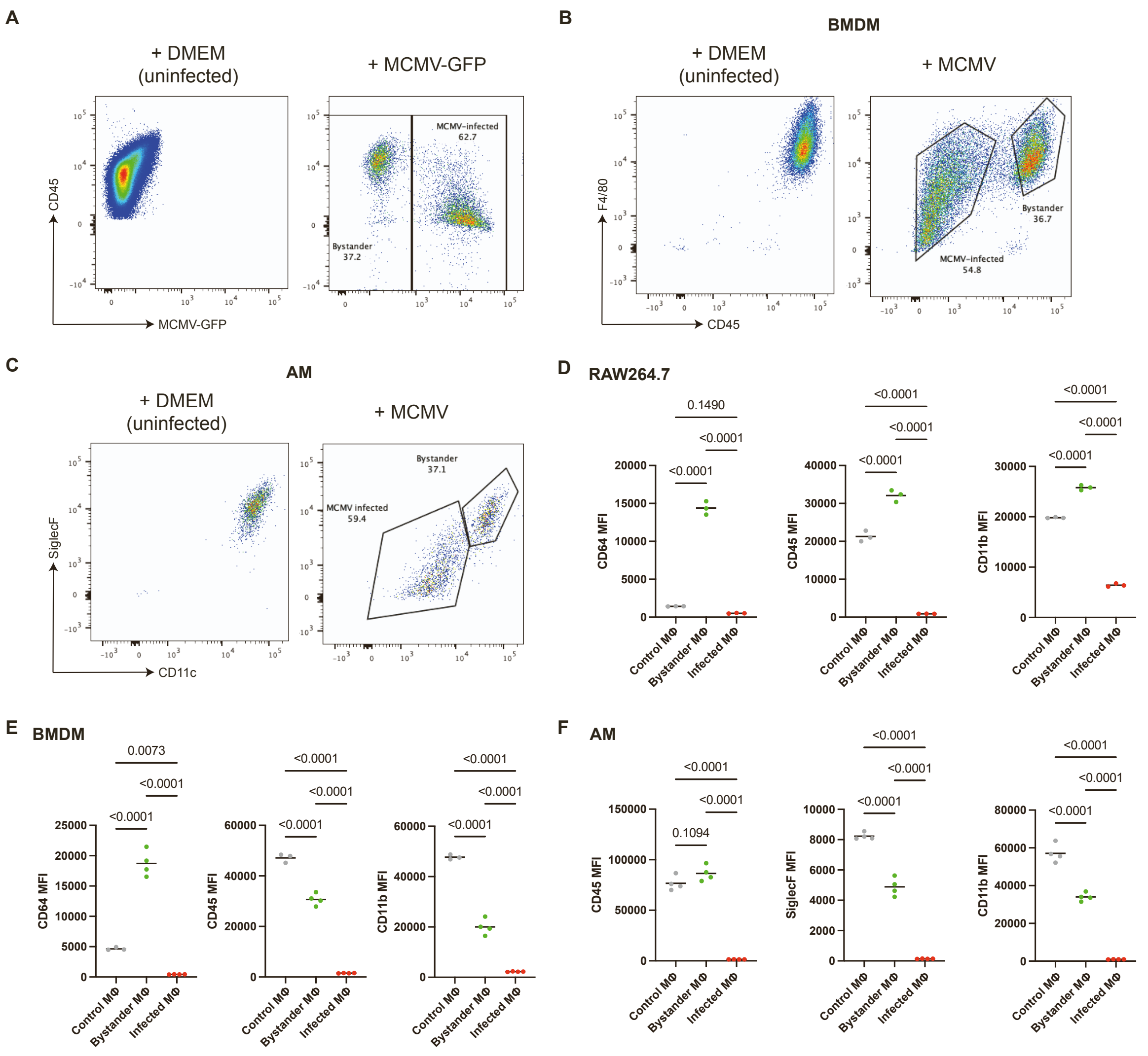

Figure S1. The influence of MCMV infection on the expression of cell surface markers in macrophages, related to Figure 1. Flow cytometry plots showing the expression of CD45 in RAW264.7 either left untreated with MCMV-GFP or treated with MCMV-GFP. Representative flow cytometry plots showing the definition of uninfected (control), bystander and MCMV-infected BMDMs (B) and AMs (C). Macrophages were either left untreated with MCMV (+DMEM) or treated with MCMV (+MCMV). The expression of phenotypic surface markers in control, bystander and MCMV-infected RAW264.7 (D), BMDMs (E) and AMs (F). The line represents the mean value. One-way ANOVA with Tukey's multiple comparison test was used to compare the expression of activation markers between different populations. Grey dots represent macrophages left untreated with MCMV, green dots represent bystander macrophages, and red dots represent MCMV-infected macrophages. *n* = 3-4 biological replicates per group. The multiplicity of infection (MOI) of MCMV was 2 for BMDMs and 1 for AMs. The MOI of MCMV or MCMV-GFP was 10 for RAW264.7. The flow cytometry was performed two days post-MCMV infection.

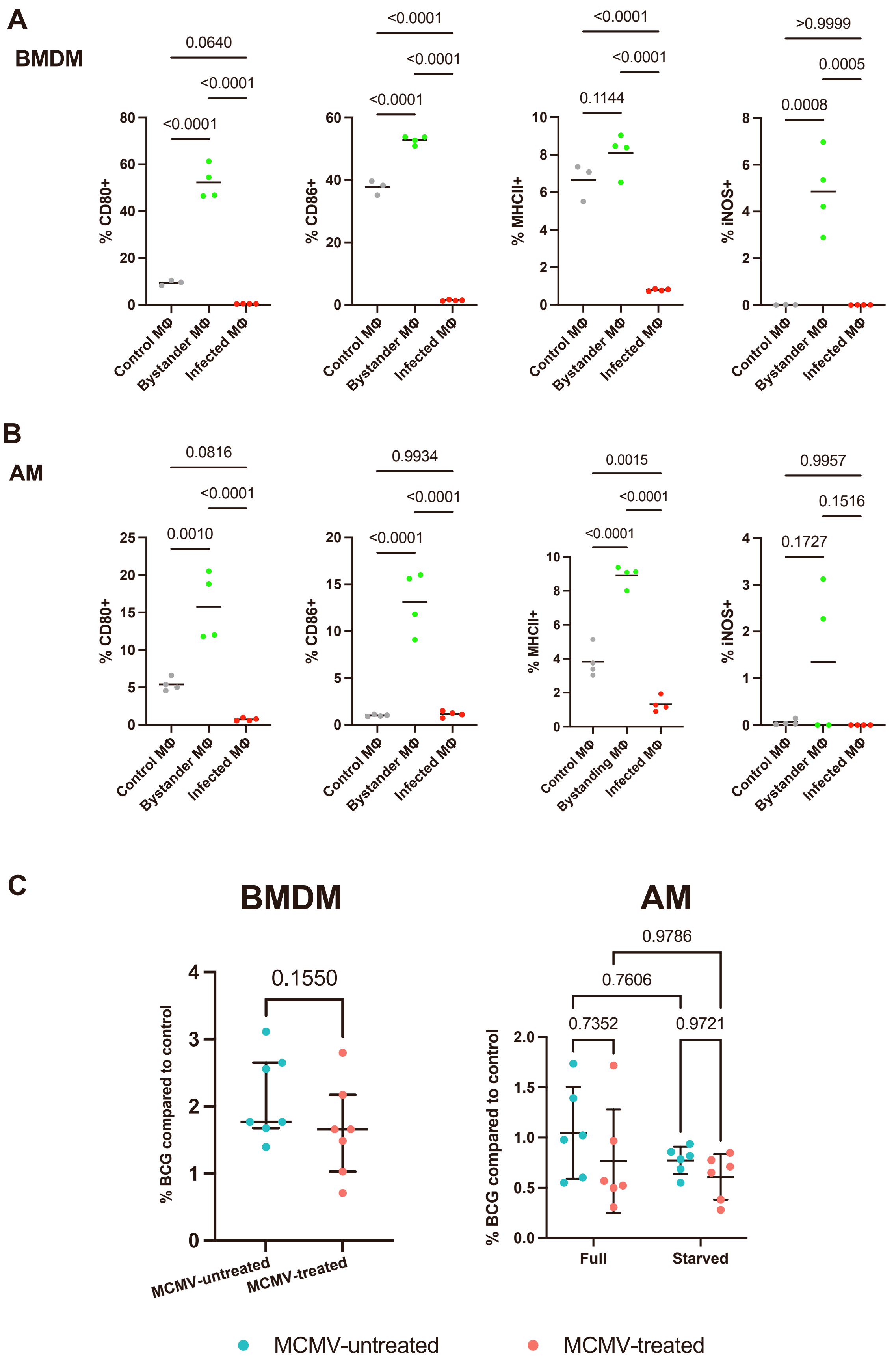
A
BMDM
B
AM
C
BMDM
AM
% CD80+
% CD86+
% MHCII+
% iNOS+
Control MΦ
Bystander MΦ
Bystanding MΦ
Infected MΦ
0.0640
<0.0001
0.1144
>0.9999
0.0005
0.0008
0.0816
0.0010
0.9934
0.0015
0.9957
0.1516
0.1727
% BCG compared to control
0.1550
MCMV-untreated
MCMV-treated
0.9786
0.7606
0.7352
0.9721
Full
Starved

Figure S2. The influence of MCMV infection on the expression of activation markers and killing of mycobacteria in macrophages, related to Figure 1 and 2. The expression of CD80, CD86, MHC-II and iNOS in BMDMs (A) and AMs (B) after MCMV infection. The line represents the mean value. One-way ANOVA with Tukey's multiple comparison test was used to compare the expression of activation markers between different populations. Grey dots represent macrophages left untreated with MCMV, green dots represent bystander macrophages, and red dots represent MCMV-infected macrophages. (C) Survival of BCG in BMDMs and AMs after MCMV infection. The BCG survival was normalised to a control group of macrophages (MCMV-treated and MCMV-untreated) infected with BCG, which were harvested immediately after a 1-h incubation to account for the difference in the phagocytosis rate. BMDM: The Mann-Whitney test was used to compare the survival of BCG between MCMV-treated and MCMV-untreated BMDMs under full nutrition. The error bar indicates the median value with the interquartile range (IQR). The upper whisker extends to the largest value no further than 1.5 × IQR from the hinge, and the lower whisker extends from the hinge to the smallest value at most 1.5 × IQR from the hinge. AM: Full represents the survival of BCG in macrophages under full nutrition. Starved represents the survival of BCG in macrophages when autophagy is induced. Two-way ANOVA with Tukey's multiple comparison test was used to compare the survival of BCG between different conditions. The error bar indicates the mean value with standard deviation (SD). n = 3-4 (A and B) or 6 (C) biological replicates per group. The MOI of MCMV was 2 for BMDMs and 1 for AMs. The flow cytometry, phagocytosis and mycobacterial killing assays were performed two days post-MCMV infection.

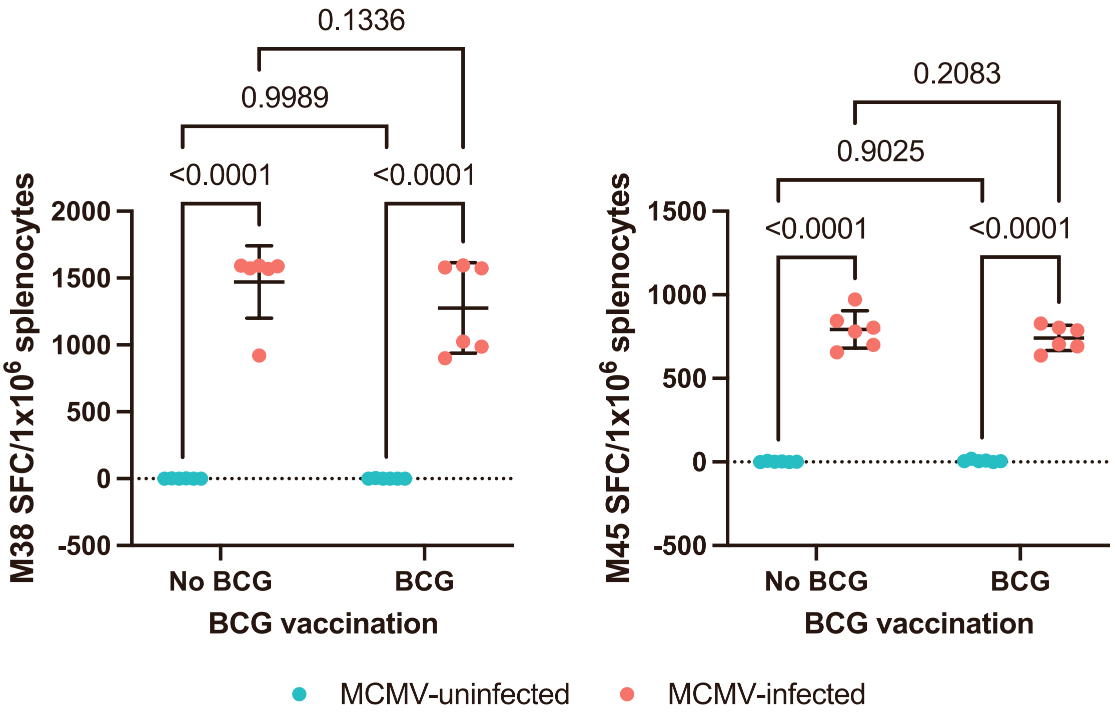

Figure S3. T cell response of splenocytes to immunodominant MCMV peptide (M38 and M45) following MCMV infection, related to Figure 3. M38- (left) and M45 (right)-specific T cell response of splenocytes from BCG-unvaccinated MCMV-uninfected, BCG-unvaccinated MCMV-infected, BCG-vaccinated MCMV-uninfected, and BCG-vaccinated MCMV-infected mice. The M38- and M45-specific T cell response was presented as the number of IFN-γ spot forming cells (SFC) per 10^6 splenocytes. No BCG means no BCG vaccination of the mice. Red dots represent MCMV-infected mice. Blue dots represent MCMV-uninfected mice. Uncorrected two-way ANOVA was used to compare the T cell response between different groups under each condition. The error bar indicates the mean value with SD. *n* = 6 mice per group. A schematic overview of the experimental design is shown in Figure 3A.

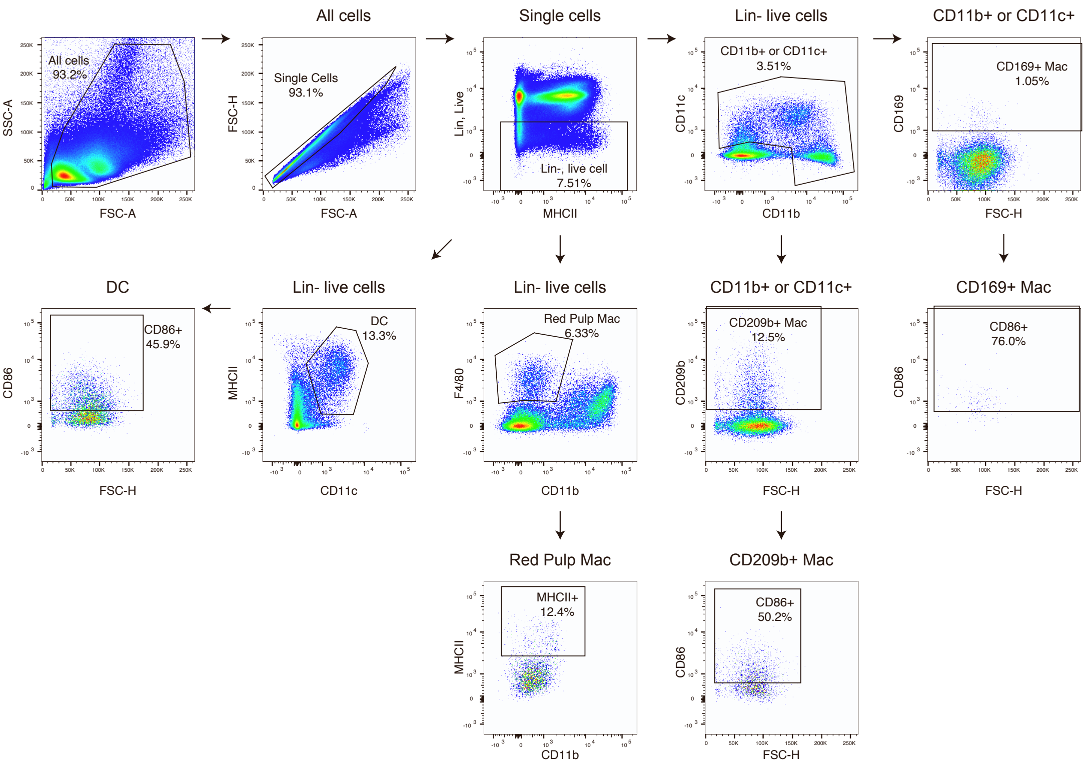

Figure S4. Gating strategy spleen DCs and macrophages and the expression of activation markers, related to Figure 4. Mice that had not been vaccinated with BCG underwent the procedures as described in the Figure 3. The expression of CD80, CD86, MHC-II and iNOS was assessed on CD209b$^+$ macrophages, CD169$^+$ macrophages and red pulp macrophages. The expression of CD80, CD86 and iNOS was assessed on DCs. The identification of DCs and macrophages was based on previous studies [S1,S2]. CD209b$^+$ Mac: CD209b$^+$ macrophages or MZMs; CD169$^+$ Mac: CD169$^+$ macrophages or MMMs; Red Pulp Mac: red pulp macrophages; Lin: lineage markers, including CD3, CD19, CD49d and Ly6G.

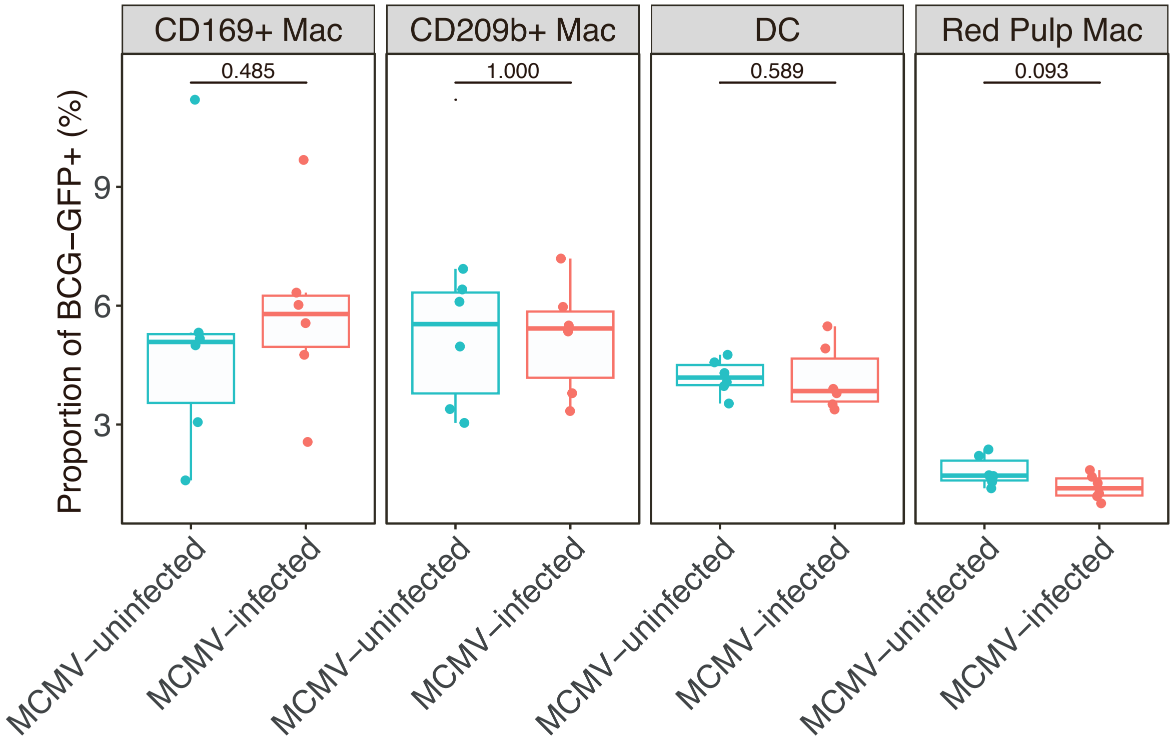

Figure S5. The influence of MCMV infection on the phagocytosis rate of BCG-GFP in DCs and macrophages from the spleen, related to Figure 4. The proportion of BCG-GFP+ cells in DCs, CD169^{+} macrophages, CD209b^{+} macrophages and red pulp macrophages in the spleens of MCMV-uninfected and MCMV-infected mice. Frozen splenocytes were thawed and left in the incubator for 4-h with BCG-GFP at MOI of 1 before staining. The Mann-Whitney test was used to compare the proportions between the two groups. *P*-values were not adjusted for multiple testing corrections. The boxplot indicates the median value with the IQR. The upper whisker extends to the largest value no further than 1.5 × IQR from the hinge, and the lower whisker extends from the hinge to the smallest value at most 1.5 × IQR from the hinge. *n* = 6 mice per group. The gating strategy of DCs, CD169^{+} macrophages, CD209b^{+} macrophages and red pulp macrophages was the same as that in Figure S4.

Table S1: Formulation of medium and buffers, related to STAR Methods

Name	Composition
R0 medium	RPMI 1640 + 2 mM L-glutamine + 100 U/ml penicillin-streptomycin
R10 medium	R0 medium + 10% fetal calf serum (FCS)
R2 medium	R0 medium + 2% FCS
ACK lysis buffer	8.29g NaCl + 1g$KHCO_3$ + 37.2g Na_2EDTA in a total of 1L of distilled water. pH 7.2-7.4
R10 medium (HEPES, no antibiotics)	RPMI 1640 with HEPES + 10% FCS + 2 mM L-glutamine
M10 medium	Minimum essential medium (MEM) + 10% FCS + 2 mM L-glutamine + 100 U/ml penicillin-streptomycin

D2 medium	Dulbecco's MEM (DMEM) + 2% FCS + 2 mM L-glutamine + 100 U/ml penicillin-streptomycin
MEM (plaque assay)	2×MEM + 2% FCS + 100 U/ml penicillin-streptomycin
1:1 CMC/MEM (plaque assay)	1 volume of 2% carboxymethyl cellulose (CMC) and 1 volume of MEM (plaque assay)
D10 medium	DMEM + 10% FCS + 2 mM L-glutamine + 100 U/ml penicillin-streptomycin
Macrophage growth medium	D10 medium (20 ng/ml M-CSF and 20 ng/ml GM-CSF were added for BMDMs and AMs, respectively. R10 medium was used for AMs.)
MCMV infection medium	D2 medium (20 ng/ml M-CSF and 20 ng/ml GM-CSF were added for BMDMs and AMs, respectively. R2 medium was used for AMs.)

Supplementary references:

S1. Fujiyama, S., Nakahashi-Oda, C., Abe, F., Wang, Y., Sato, K., and Shibuya, A. (2019). Identification and isolation of splenic tissue-resident macrophage sub-populations by flow cytometry. Int. Immunol. *31*, 51–56. https://doi.org/10.1093/intimm/dxy064.

S2. Perez, O.A., Yeung, S.T., Vera-Licona, P., Romagnoli, P.A., Samji, T., Ural, B.B., Maher, L., Tanaka, M., and Khanna, K.M. (2017). CD169 $^{+}$ macrophages orchestrate innate immune responses by regulating bacterial localization in the spleen. Sci. Immunol. *2*, eaah5520. https://doi.org/10.1126/sciimmunol.aah5520.
